# ID 203 - Uso da Fronteira de Eficiência e Benefício Líquido na Avaliação de Tecnologias em Saúde: um estudo aplicado à esclerose múltipla no Brasil

**DOI:** 10.5327/2237-9622.2025.v34s1.203

**Published:** 2025-11-25

**Authors:** Bruno Monteiro Barros, Marcelo Goulart Correia, Bernardo Rangel Tura, Carlos Alberto da Silva Magliano

**Keywords:** fronteira de eficiência, benefício líquido, esclerose múltipla, custo-efetividade

## Abstract

**Introdução:**

A Avaliação de Tecnologias em Saúde (ATS) é utilizada como ferramenta na tomada de decisões para a incorporação de novos tratamentos nos Sistemas Único de Saúde (SUS). Tradicionalmente, a análise de custo-efetividade é a principal ferramenta utilizada para comparar tecnologias que são submetidas ao processo de incorporação na Comissão Nacional de Incorporação de Tecnologias no Sistema Único de Saúde (Conitec). No entanto, essa abordagem apresenta limitações ao avaliar múltiplas intervenções simultaneamente. A fronteira de eficiência e a análise de benefício líquido surgem como ferramentas complementares para esse tipo de análise.

O presente trabalho tem como objetivo demonstrar a aplicação dessas ferramentas na avaliação dos tratamentos utilizados para esclerose múltipla remitente recorrente (EMRR) no Brasil. O objetivo geral é mostrar como essas técnicas podem complementar a análise de custo-efetividade, fornecendo uma visão mais abrangente para a tomada de decisão em saúde.

**Método:**

Um modelo de Markov foi construído a partir da perspectiva do SUS para conduzir as análises econômicas. O modelo considerou a taxa anualizada de surtos e a progressão da incapacidade sustentada por seis meses, medidos de acordo com a Escala Expandida do Estado de Incapacidade (EDSS). O horizonte temporal considerado foi de vida toda. O das tecnologias comparadas ao placebo foi obtido por meio de uma metanálise em rede. A efetividade foi medida por qualidade ajustada por anos de vida (QALY) e custos obtidos por meio de fontes governamentais e por um estudo de custeio da doença no Brasil. A análise de benefício líquido e a fronteira de eficiência foram aplicadas para comparar 14 medicamentos aprovados no Brasil para o tratamento da EMRR.

**Resultados:**

A análise de custo-utilidade revelou que, entre os 14 medicamentos avaliados, 12 foram dominados por alentuzumabe e teriflunomida. Uma fronteira de eficiência foi estabelecida entre esses dois medicamentos, com uma razão de custo-efetividade incremental (ICER – do inglês, ) de R$ 41.159,38 por QALY. Na análise de benefício líquido, a teriflunomida apresentou os melhores resultados. Na análise de sensibilidade probabilística, a maioria dos medicamentos mostrou ICERs abaixo do limiar de custo-utilidade adotado no Brasil (R$ 40.000,00 por QALY).

**Conclusão:**

A abordagem de tratamento da EMRR compreendida neste estudo foi a de tratamento intensivo precoce, na qual todos os medicamentos são analisados em uma mesma linha de tratamento. Essa ainda pode ser uma limitação para uma aplicação imediata no SUS, que prevê tratamento escalonado para EMRR. A fronteira de eficiência e a análise de benefício líquido fornecem uma análise complementar à custo-efetividade, auxiliando na definição da utilidade econômica dos tratamentos disponíveis. Os resultados indicam que o uso dessas ferramentas pode funcionar como um marco na definição do preço de outros medicamentos submetidos para futuras incorporações ao SUS.

